# A model comparison reveals dynamic social information drives the movements of humbug damselfish (*Dascyllus aruanus*)

**DOI:** 10.1098/rsif.2013.0794

**Published:** 2014-01-06

**Authors:** R. P. Mann, J. E. Herbert-Read, Q. Ma, L. A. Jordan, D. J. T. Sumpter, A. J. W. Ward

**Affiliations:** 1 Mathematics Department, Uppsala University, Uppsala, Sweden; 2 Ecology and Genetics Department, Uppsala University, Uppsala, Sweden; 3 School of Biological Sciences, University of Sydney, Sydney, New South Wales, Australia; 4 School of Ecology and Evolution, University of South Wales, Sydney, New South Wales, Australia

**Keywords:** collective behaviour, collective decision-making, Bayesian model selection, social information

## Abstract

Animals make use a range of social information to inform their movement decisions. One common movement rule, found across many different species, is that the probability that an individual moves to an area increases with the number of conspecifics there. However, in many cases, it remains unclear what social cues produce this and other similar movement rules. Here, we investigate what cues are used by damselfish (*Dascyllus aruanus*) when repeatedly crossing back and forth between two coral patches in an experimental arena. We find that an individual's decision to move is best predicted by the recent movements of conspecifics either to or from that individual's current habitat. Rather than actively seeking attachment to a larger group, individuals are instead prioritizing highly local and dynamic information with very limited spatial and temporal ranges. By reanalysing data in which the same species crossed for the first time to a new coral patch, we show that the individuals use static cues in this case. This suggests that these fish alter their information usage according to the structure and familiarity of their environment by using stable information when moving to a novel area and localized dynamic information when moving between familiar areas.

## 1. Introduction

Animals frequently use social information in making decisions [1–4], but how does information transfer between group members? Although a human group might set up a highly structured voting procedure to allow for preference-pooling [5], animals must typically rely on behavioural cues to gain information about the decisions and actions of others. Theoretical and experimental studies of animal groups have shown that information transfer can be explained as the result of many simple local interactions between close neighbours [6–10]. In theory, such neighbour-following behaviour can explain collective decision-making [11,12].

Despite the fact that simulation models can reproduce many global-level aspects of the outcome of decision-making experiments, this does not imply that we know the underlying cues used by individual animals [13]. For example, quorum models have been applied in modelling the decisions of fish about whether to move to the left or right in a Y-maze [14–16]. In these models, the proportion of fish committing to move left is a sharply increasing nonlinear function of the number which have already committed to this choice [17]. A convincing theory supporting quorum-like responses has been developed based on a Bayesian analysis of what an individual within the group should believe based on the actions of others [18,19]. However, quorum responses are consistent with many different types of cue-following behaviour [11,20]. Similarly, mechanisms akin to voting have been observed in relatively small groups where all members can observe each other [21–23]. But in these groups, how local is the range of communication between individuals both spatially and temporally? If interactions are local, what specific cues do animals pay attention to? Identifying which cues individuals respond to is an important step in understanding how and why animals make these decisions.

Determining the nature of these cues is however non-trivial. When individuals respond to the cues produced by nearby conspecifics, then the decision by one individual to make a particular choice or engage in a particular activity affects the choice of others. This decision, in turn, affects how successive individuals will chose one or the other options. These decision sequences make it difficult to identify the cues used by individuals, because different elements of the social environment are highly correlated over time. For example, consider a situation where at time *t* a focal fish has one neighbour to its left and one to its right and then shortly afterwards, at time *t* + 1, both of its neighbours have moved to be on its left-hand side. We then observe at time *t* + 2 that our focal fish turns left. The question is whether it is the *dynamic* movement of the neighbour between timesteps *t* and *t* + 1 or whether it is the *static* arrangement of neighbours at time *t* + 1 which are critical in determining the focal fish turning at *t* + 2. In other words, whether each individual pays attention to the current positions/behaviours of each available conspecific or gives greater weight to recent *changes* of behaviour. This sketched example does not provide a sufficient level of description to address this question directly, but it exemplifies the general problem of correlation of cues. Because static and dynamic cues can be highly correlated, it is possible that responses to dynamical cues may produce a significant relationship between decisions and static information, and vice versa. Furthermore, a continuum exists between static and dynamic responses which will ultimately depend on the memory window of the animal. Animals may use both these forms of information to inform their decisions. Teasing apart this correlation and identifying the sources of information and cues used by individuals is the challenge we address here.

Previous studies investigating the role of social information in a variety of species have focused on the static cues provided by conspecifics at the moment when an individual makes its decision to move, in groups of fish [14,15,24], mammals [21,25,26], birds [27,28] and insects [29,30]. Such static information can, for example, take the form of the positions of conspecifics, the number of individuals standing/sitting, the amount of noise being made by other individuals or the directions of their gazes [21]. A smaller number of studies have investigated cues more akin to dynamical information, e.g*.* [31,32], though the strong correlation of dynamic and static information in these cases makes it difficult to identify which cue is more important. However, no studies have empirically investigated the relationship between dynamical and static cues in animal groups in contexts where these may provide conflicting information, and developed a methodology for isolating the primary stimuli the animals respond to in their decision-making.

**Figure 1.**
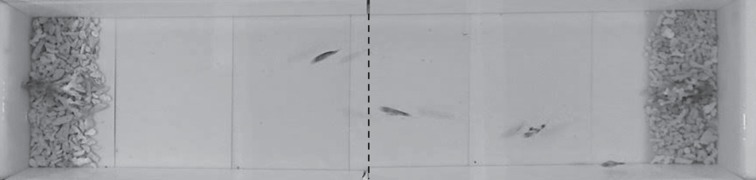
Image of the experimental arena showing the location of the two coral patches with a piece of coral skeleton in the centre of each patch. The image shows three fish on the right side of the tank and about to cross into the left side, while another fish has just crossed over and into the left side. The dashed line indicates the previous position of the central divider (and centre of the tank) which was removed after 5 min of fish acclimation, allowing the fish to move between patches.

In this study, we investigate how social interactions and behavioural mimicry lead to decisions in the groups of humbug damselfish (*Dascyllus aruanus*). In particular, we examine the movements of these fish between two coral patches in an experimental arena (figure 1). We took advantage of these typical repetitive movement decisions to investigate whether individual movements between patches were influenced by the number of other fish that had crossed between patches or by those that had just crossed. As predation rates are high for small reef fish and predator attacks are more successful when fish are exposed from their refuges [33,34], deciding when it is safe to move between coral patches is particularly important. Humbug damselfish are a tropical pomacentrid fish which live in discrete social groups composed primarily of unrelated individuals [35]. Groups of these fish are stable over time and fish preferentially associate with familiar rather than unfamiliar individuals [36]. They live on branching acroporan and pocilloporan coral colonies [37,38] which they use as a refuge from predators [39]. They show strong site fidelity with respect to their home coral colony and may have multiple coral patches within their territories which fish move between, both on their own and in the groups (JE Herbert-Read and AJW Ward 2011, personal communication). Fish rarely stray more than 1 m away from these home corals [40].

We investigate whether static/positional information [14,15,17], dynamic/movement information or both forms of information are more important in driving individual decisions to move. In particular, we compare our experiments with recent work by Ward *et al.* [41]. This study demonstrated that the probability for this species of damselfish to leave a relatively safe environment increases linearly with the number of conspecifics that have already done so, suggesting a static rule for movement decisions. However, this earlier work and the current observations are subject to the potential confounding of static and dynamic information described earlier. To account for this, here we take a Bayesian model selection approach [13,24,32,42–44] to identify the cues each individual uses to overcome this problem, combined with an experimental set-up that creates potential conflicts between dynamical and static information. We use simulation studies to determine which of our models were better at explaining both the observed fine-scale movement dynamics and the large-scale distributions of fish movements between the two coral patches. We also determine whether some individuals were more likely to initiate and lead crossings and whether hierarchical leader–follower relationships existed when groups crossed between patches.

## 2. Results

### 2.1. Distribution of fish and their movement between coral patches

**Figure 2.**
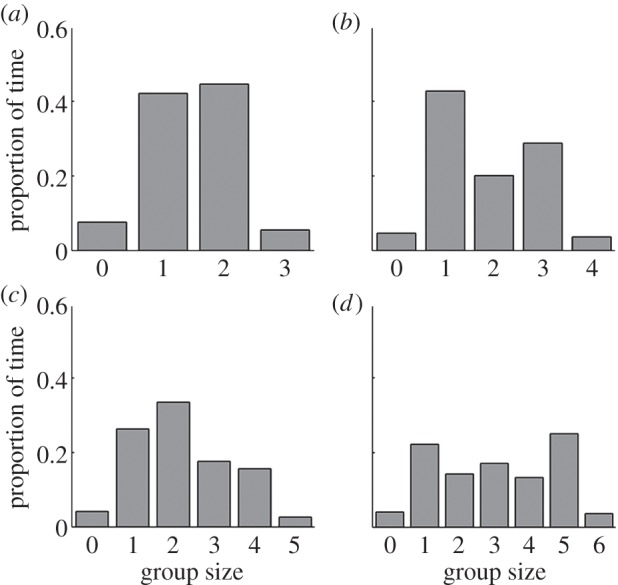
Experimental results show the proportion of time different numbers of fish were found on the left side of the tank for each group size. Results from group sizes of (*a*) three, (*b*) four, (*c*) five and (*d*) six. In all cases, the most common configuration is with approximately half of the fish on each side of the tank, suggesting a potentially asocial dynamic. Our model selection results demonstrate that the fish do obey social cues, but this social response is too weak to consistently keep all the fish together on one side of the tank.

**Figure 3.**
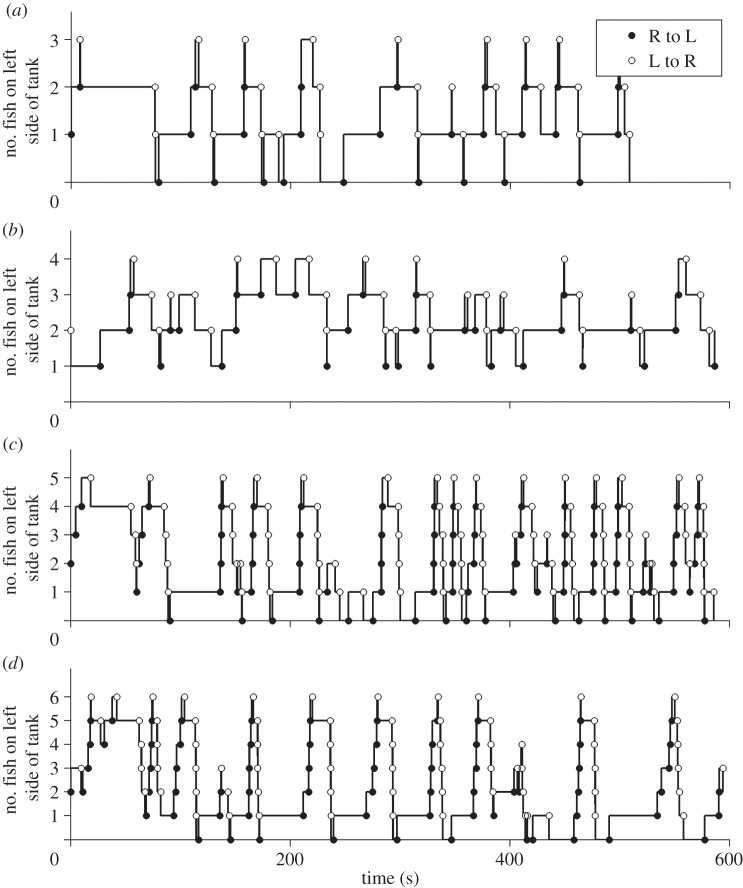
Examples of recorded crossings in experiments of different group sizes. Each panel shows the number of fish on the right-hand side of the tank over the duration of the experiment for group sizes of (*a*) three, (*b*) four, (*c*) six and (*d*) six fish. Black marks indicate times where a fish crossed from the left-hand side to the right-hand side, white marks where a fish crossed from the right-hand side to the left-hand side.

Fish spent significantly more time on the coral patches than in any other region of the arena, indicating strong bias to associate with either coral patch (group sizes of three: binomial test, *N* = 16, *n* = 15, *p* < 0.001; group sizes of four: binomial test, *N* = 16, *n* = 14, *p* < 0.01; group sizes of five: binomial test, *N* = 11, *n* = 11, *p* < 0.001, group sizes of six: binomial test, *N* = 14, *n* = 11, *p* = 0.029). Crossings to the left of the tank were as frequent as crosses to the right side of the tank indicating no side preference in the arena (*N* = 4433, *n* = 2207, two-sided sign test: *p* > 0.78 in all trials). The distribution of the proportion of time different numbers of fish were on the left-hand side of the arena generally followed an n-shaped distribution (figure 2), where all individuals were generally not found together on one side of the arena. However, it was clear that individuals in the arena generally tended to cross in groups (figure 3). Indeed, the number of fish in the crossing group was often equal to the total number of fish that could have potentially crossed (figure 5*b*), indicating that all fish that were on one side of the arena generally tended to cross together. Why then, were all group members not always found together? This can be explained by our model classifications in the following.

### 2.2. Model comparisons

**Figure 4.**
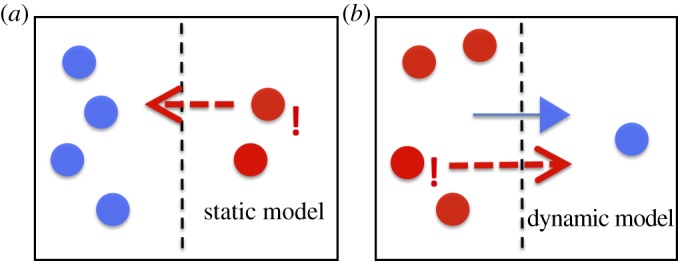
An illustration of the difference between static and dynamic models. (*a*) In the static model, the fish on the right are individually more likely to be the next movers (red), because they are in the smaller group and are attracted to the larger group. (*b*) In the dynamic model the fish on the left are individually more likely to move despite being in the larger group, because they would be following the last mover (shown by a triangle).

If the movement of the individual fish between the two coral patches is at least partially controlled by social factors such as attraction to other individuals and leader–follower relations, then those movements should be predictable to some degree from the current positions and recent movements of the other fish. We therefore constructed models to predict these movements using a number of alternate hypotheses for those social interactions. As well as a null hypothesis with no social interactions, we chose to investigate two primary classes of model. *Static* models predict that the propensity of an individual fish to cross depends on the current spatial configuration of the group, i.e. how many fish are on each side of the tank. Alternatively, *dynamic* models predict that this propensity depends on the recent movements of the fish, i.e. which fish have recently crossed the tank and in which direction. Figure 4 illustrates this difference. Figure 4*a* shows an example of a static model; the fish highlighted in red are more likely to move next, because they are attracted to the larger group on the other side of the arena. By contrast, figure 4*b* shows a dynamic model, where the highlighted fish are more likely to move because they would be following the last mover (shown by a triangle). Within these two classes, the propensity of individuals to respond to the positions or movements of the other fish can take a variety of forms, which are discussed in the electronic supplementary material, along with precise mathematical descriptions of each model.

**Figure 5.**
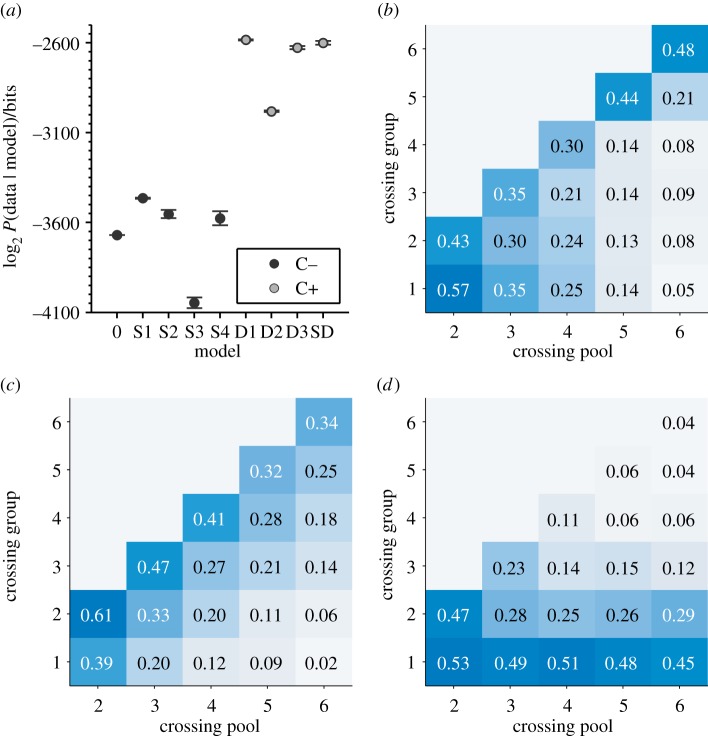
Large-scale and fine-scale model comparison, combined over all group sizes. (*a*) Log-marginal-likelihoods evaluated for the seven tested models. Model D1 (‘follow last mover’) is the optimal selected model, with a large likelihood ratio compared with all other models. Within static models (S1–4), model S1 (‘binary response’) is the best fit. Models marked as black or grey circles were respectively inconsistent or consistent in reproducing the large-scale patterns of the data (*b–d*); (*b*) experimental results showing the proportion of time a crossing group of size *n* crossed the arena from the potential number of fish (crossing pool) that could have crossed (i.e*.* the number of fish that were initially present on the side from which the crossing was initiated.) In each case, the most probable movement is all the available fish from the pool crossing together, indicating a strong preference to follow the movements of local conspecifics. (*c*) Large-scale movement groups sizes obtained from simulation of the best-fit dynamic model (D1), showing consistency with the experimental pattern. (*d*) Large-scale movement groups sizes obtained from simulation of the best-fit static model, S1, showing inconsistency with the experimental pattern. See the electronic supplementary material for a breakdown of results by different group size experiments and for full model details. (Online version in colour.)

Figure 5 shows the results of our model comparison. Figure 5*a* shows the log-marginal-likelihoods, log_2_*P*(*𝒟/M_i_*), for all the different models, evaluated over the complete dataset of all experiments *𝒟*. The models are organized into the two principal categories of static (*S*) or dynamic (*D*), and within these categories, each numbered model represents a different response to the primary static or dynamic cue (full details given in the electronic supplementary material text). Overall, the best model for all group sizes is model D1, which predicts that individual fish are more likely to move if they follow the single last mover. Specifically, if the last crossing was from left to right, then individuals on the left will be individually more likely to move next, and vice versa. Within the static models, the overall best is model S1, the binary response decision model, where fish are more likely to move to the larger group, independent of the difference in group sizes. The difference in the likelihood between the static models is small compared with the difference between all the static models and the dynamic models. We found that combining the optimal static and dynamic models did not improve on the performance of model D1, indicating that any predictive power from the static configuration likely comes from its correlation to recent movements of the fish. The superior performance of dynamic models is repeated across group sizes when experiments with different numbers of fish are analysed separately (see the electronic supplementary material, figure S1).

We assessed the probability of different models by analysing the movements of individual fish. However, it is a necessary condition of any model that it can reproduce the large-scale patterns in the data, because we aim to understand how these emerge from the interactions between individuals (see [45]). Therefore, using the rules of interaction specified by these models, we simulated crossing events and investigated whether each model was adequate in reproducing the larger-scale dynamics of the system. In particular, we asked whether these models reproduced the observation that the crossing group size tended to equal the number of fish that could have potentially moved from that side of the tank (shown in figure 5*b*). We found that only the dynamic models, where individuals only pay attention to local changes, reproduced crossing group sizes (figure 5*c*). On the other hand, the static models were inadequate at reproducing such large-scale patterns of the data (figure 5*d*). Therefore, on both the fine- and large-scale the dynamical models proved better at describing the decisions that produce the observed crossing behaviour. The models evaluations in figure 5*a* are colour-coded according to their consistency with this large-scale behaviour, with grey markers indicating consistency (C+) and black markers inconsistency (C−). Figure 5 shows results aggregated across different group sizes, see the electronic supplementary material, figures S1–S4 for group-size-specific results.

Successive moves between coral patches were more likely to be in the same direction (60%) than not (40%). However, when the time between successive moves was more than 3.5 s crossings were more likely to be in opposite directions than expected from these averages (see the electronic supplementary material, figure S5). This provides further evidence that short-term temporal information (D1 model) is more important in driving fishes’ decisions to move between patches rather than the other forms of information described in the alternate models. We considered whether fish might switch strategies to using spatial information if none immediately followed the recent movement of a conspecific. To do this, we used the subset of data with longer intervals between successive crossings to investigate whether the static models were better at describing fishes’ movements between patches when there were longer delays (more than 3.5 s) between successive crossings. However, because most movements occur within 3.5 s of the previous crossing (see the electronic supplementary material, figure S6), there was insufficient data in this subset to confidently establish differences between different models. The increased probability of moves in opposite directions after 3.5 s is likely the result of many longer intervals occurring when all fish are on the same side of the tank, when the next move is necessarily in the opposite direction. These cases do not contribute to our model selection.

In a similar recent experiment involving movements between a refuge area and open water, Ward *et al.* [41] identified a positive linear relationship between the probability that an individual would leave the refuge and enter the open water area and the number of conspecifics already in the open water. A similar relationship also held for the probability to return to the refuge. A rule of following the last mover could potentially explain these observations, because the number of conspecifics in either environment is strongly correlated to the direction of the last movement. We wanted to see whether our model selection methodology would support the conclusions of Ward *et al.* [41], or alternatively indicate a common behaviour rule for both experiments.

**Figure 6.**
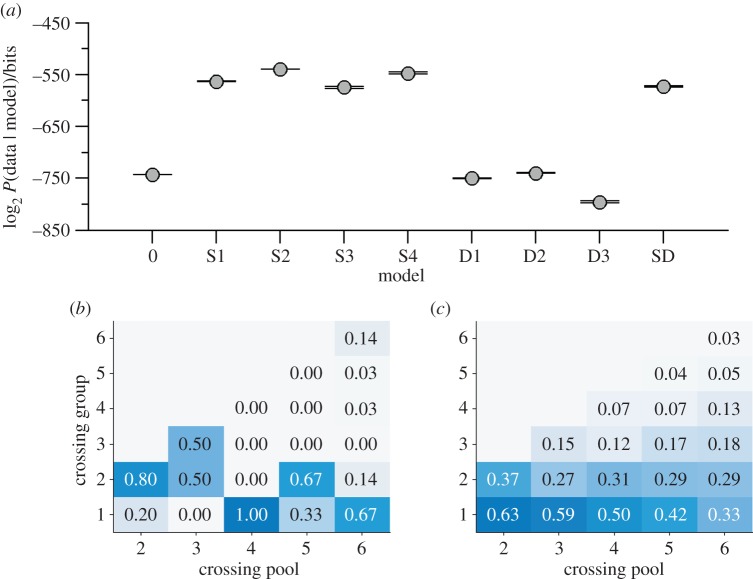
Model comparison on experimental data from Ward *et al.* [41]. (*a*) Log-marginal-likelihoods evaluated for the seven tested models, combined across all experiments and all group sizes. Model S2 is the optimal selected model, indicating a linear response to the difference in the number of conspecifics in the current or alternative environments. (*b*) Large-scale view of the experimental data, showing the bout sizes (number of fish crossing together in one direction) as a function of the potential pool of movers. Most bouts involve only one or two fish. (*c*) The distribution of bout sizes in simulations of the best-fit model S2, showing a similar pattern of small bout sizes. (Online version in colour.)

To test this, we applied our models to the single coral environment. Instead of two identical sides of the tank, we aim to predict movements between the refuge and the open water, but otherwise the models are identical. Testing these models on the data of individual movements to and from the open water we see in figure 6*a* that the static models which use the positions of conspecifics, either in the refuge or the open water, outperform the dynamic models based on the directions of the last mover(s). The linear model (S2) is the most probable of these, supporting the conclusions of [41] and showing a different pattern of behaviour to that seen in this study. It should be noted that the Bayesian decision-making model (S4) [18] performs similar to the linear model, because this model is approximately linear in this group size regime where the difference in the number of conspecifics is usually small.

Figure 6*b* shows the experimental distribution of ‘bouts’ as a function of the potential crossing pool in the Ward *et al.* study in a similar manner to figure 5*b*. Here, we can see that the large-scale pattern of movements is also different in the Ward *et al.* study from our own—the most common bout sizes are small, involving only one or two fish. The distribution of bout sizes in simulations of model S2 mimic this pattern in figure 6*c*, lending further support to this model in this context.

### 2.3. Leadership and hierarchical movement decisions

Individuals that crossed more times by themselves were also the individuals that were more likely to lead other fish when crossing in groups (Pearson *ρ* = 0.16, *p* = 0.01). These fish were also more likely to be the larger individuals (Pearson *ρ* = 0.15, *p* = 0.01) in the group. We also found tentative evidence that hierarchical leader–follower dynamics existed when all group sizes were analysed together (Fisher omnibus test and Kendall linearity coefficient Monte Carlo test *χ*^2^ = 93.9, d.f. = 68, *p* = 0.02) but this result did not hold when group sizes were analysed separately (see the electronic supplementary material for details).

## 3. Discussion

Our model comparison approach revealed that humbug damselfish responded to the local movements of neighbours and made their decisions to move according to ‘dynamic’ information. They did not use static or global information based on the numbers of fish on either coral patch to inform their decisions to move. Observing the dynamic behaviours of neighbours allows individuals to gather information based on recent events rather than relying on static information from previous decisions that may be unreliable under current environmental conditions [46]. This is important as in some cases, changing environmental variables such as the distribution of food, predators or mates, can quickly alter the benefits afforded by different areas available to move to [47]. In such situations, the relatively small amounts of ‘up-to-date’ information, such as recent movements, may be preferable to the more robust but slower changing information given by the spatial distribution of conspecifics. Our results suggested a timescale for the salience of dynamic information of approximately 3.5 s. Longer intervals between moves were associated with an increased probability of movement in opposite directions, though many of these longer intervals occurred when the fish were all on the same side of the tank. Overall, there were insufficient data to draw conclusions about the rules of interaction after the dynamic saliency period.

This dynamic information strategy may be used under different contexts to inform animals’ decisions. Chacma baboons (*Papio hamadryas ursinus*) appear to watch the departing movements of others when deciding to move from resting sites [48]. Humans are typically more likely to start crossing a road if their immediate neighbours are already crossing [49]. Sometimes, this can subsequently lead individuals to abandon crossing events when vehicles are approaching, hinting at the disadvantage of dynamic information use in this case [49]. Many anti-predatory responses involve individuals’ rapid movements away from a predator which may act as cue informing conspecifics of a detected threat [50–52].

The different models favoured by our experimental data and that from a previous and closely related study on the same species [41] suggests that these fish change the cues they attend to in response to a different environment. The principal differences in the experimental set-up between our study and [41] are the extended nature of the tank and the repeated decision-making necessary by the fish. The longer tank may make visual contact with the other side more difficult or impossible (though we have no direct evidence for this). Meanwhile, repeated decisions and crossings of the tank may give individuals a greater personal familiarity with the environment and induce a change in behaviour. In particular, the experiments by Ward *et al.* [41] always ended once consensus was first established, whereas, in our experiment, consensus repeatedly emerges and is broken. Although the fish are often all on the same side of the tank, the continued exploration of both sides by individual fish means that this ‘consensus’ is not maintained indefinitely, meaning that the fish repeatedly have the chance to respond to both consensus and divided group situations.

There may be more complex mechanisms determining whether a crossing is initiated as individuals assess whether others want to, or are about to, leave the coral [41]. This would represent a pre-crossing stage which is not explicitly included in our modelling methodology. This pre-crossing stage, where fish assess whether there is consensus for leaving the coral, may involve ‘static’ spatial information, such as the number of fish currently on or off the coral, as in Ward *et al.* [41], which would be akin to the static models we have described in this paper, but such information would have to be localized to each side of the tank individually, because we have shown that crossing probabilities do not depend on the relative number of fish on each side of the tank.

Leadership can emerge by individuals having higher propensities to initiate movements or lower propensities to abandon these initiations [53]. In our groups, larger damselfish crossed more frequently by themselves than smaller individuals and were therefore, more likely to initiate crossing events which were subsequently followed by others. These larger, and therefore more dominant individuals as reported in these fish [37], emerged as leaders within these groups. This is true for other more cognitively complex animals such as rhesus macaques [54]. The similarities between leadership in these groups hints at how simple mechanisms can drive coordinated group movement in both cases. We suggest that these initiators of group movement are important in producing the dynamic information required to initiate future individuals’ crossings. Without them, crossing events are likely to be less common. Unlike primate systems where it is often difficult to manipulate groups, these fish provide an excellent system to investigate the role these dominant individuals play in producing information that drives decision-making processes in socially structured groups.

When studying collective systems, it is important to consider both the fine- and large-scale dynamics of the system and to maintain consistency between these [43,45,55]. Here, we showed that although the patterns of distribution of animals appeared weakly social, on the fine scale, the fish displayed a strong propensity to follow the movements of conspecifics. Through simulations, we shown that this fine-scale behaviour was consistent with the large-scale behaviour of the group. We have integrated these using the methodology laid out in Sumpter *et al.* [45], using a cycle of observing large-scale phenomena, proposing individual-level interactions to explain these phenomena, of which we assess the likelihood using Bayesian model selection at the fine scale, and finally checking the consistency of the selected rules with the large-scale emergent group behaviour by simulation of the selected model. Further manipulating the social cues available to individuals before and during collective decisions will provoke a wider variety of possible individual-level sensory responses, allow for selection over a wider variety of interaction models and provide intriguing insights into the decision-making process.

## 4. Material and methods

### 4.1. Experimental animals, methods and protocols

Research was carried out at One Tree Island (−23°30′26″, 152°5′25″), Great Barrier Reef, between 16–24 September 2010 and 10–14 January 2011. We collected fish by lightly anaesthetizing them using a mix of clove oil, ethanol and seawater. Fish were caught using hand nets and were transported in mesh cages allowing water flow and thus aiding the fishes’ recovery from the anaesthetic. Fish recovered from the anaesthetic within less than 3 min. We transported fish back to aquaria facilities and placed each group into its own housing tank (645 × 413 × 276 mm) with flow-through saltwater pumped in from the lagoon. In each housing tank, we placed pieces of dead coral for refuge. The fish were left to acclimate to the aquaria for at least 36 h prior to experimentation. Fish were fed flaked fish food, and zooplankton collected with seine nets ad libitum. The fish acclimated quickly to the aquaria facilities, and we did not observe any mortality over the time fish were kept in captivity (maximum 5 days). After experimentation, all fish were returned to where they were caught.

We constructed a rectangular arena (300 × 1400 × 210 mm) from 6 mm white Perspex (figure 1). At each end of the arena, we placed small pieces of coral rubble on the tank floor so that they covered an area of 330 cm^2^. We also placed a piece of coral skeleton (longest dimension 100 × widest dimension 80 mm) in the centre of the coral rubble at each end of the arena, ensuring that these two pieces were of similar size. A central divider (210 × 300 mm) made of 3 mm white opaque acyclic initially divided the two halves of the arena, but could be remotely removed using a monofilament line. The removal of this divider then connected the two halves of the arena. The arena was filled to a depth of 180 mm with seawater and was lit using 40 W fluorescent lamps.

For each trial, we randomly selected a number of fish from one of the housing tanks and placed them into the arena, ensuring that we initially had at least one fish on each side of the arena. We selected group sizes of three (*n* = 16), four (*n* = 16), five (*n* = 11) or six individuals (*n* = 14). To abide by animal ethics and National Marine Park protocols, we did not catch enough groups to only be used once. Therefore, we re-used fish between trials, but randomized group size over time and fish were never used in the same group size more than once. After fish had been in the arena for 5 min, we remotely removed the central divider, allowing fish to move between the two coral patches. We filmed trials (at 15 fps) for 10 min using a camera (Logitech Pro 9000) placed directly above the centre of the tank. After each trial, we took photos of each fish to later calculate each fish's size and then returned fish to their original housing tank. Fish were only trialled once per day with a maximum of three trials each over the course of all trials.

Please contact the corresponding author if you wish to request the original data collected for this study.

### 4.2. Distribution of fish and their movement between coral patches

Videos were imported into VirtualDub (v. 1.9.2). We point sampled nine times during each trial every 1000th frame and counted how many fish did not have any part of their body over either coral patch. Using a sign test, we asked how many trials had more fish on the coral than off the coral over the course of each trial when compared with random chance. If coral was not attractive or repelling, then by chance, only half the trials should have more fish on the coral than off the coral. This chance is based on a conservative estimate of the area of tank taken up by both coral patches and a possible attraction to the walls and corners of the tank (figure 1). We analysed different group sizes separately. We imported the images of fish into ImageJ (v. 1.36b) and determined the length of each fish (snout to base of tail) by a rule visible in each photo.

Fish frequently moved between the two coral patches in the arena. We defined a crossing (between patches) when a fish moved completely over the central line of the arena (where the divider had been) and into the other side of the arena. We recorded all crossings that happened during each 10 min trial. For each crossing, we recorded the time at which it occurred (in frames), whether it was from the left to right or right to left, and the individual identity of each fish that crossed. By recording the identity of each fish's crosses, we obtained information on the order of individual's crosses.

We then determined the proportion of time that different numbers of fish were found on each side of the tank and the time between successive moves. When individuals crossed successively in the same direction, we defined these individuals as in a single *crossing group*. In practise, our definition concludes that two fish crossing with any time duration apart, but in the same direction were in the same crossing group. As shown in the electronic supplementary material, figure S6, however, over half of all crosses occurred within 2.5 s of one another, and the electronic supplementary material, figure S5 indicates that those which were in the same direction are associated with shorter intervals. Fish that could have potentially moved in a crossing group (i.e. those fish on the side of the tank that the group moved from) were defined as the *crossing pool* for this event. We determined the relationship between the number of fish in each crossing group and their associated crossing pool sizes by calculating the frequency of different crossing group sizes for each crossing pool size.

### 4.3. Model selection

We use a Bayesian model comparison to select between these alternative explanations of the data, following the methodology of [13,43,44]. Each model gives a probability for any observed crossing event, by determining a probability that the next move will come from either the left or right-hand side of the arena (full model details are given in the electronic supplementary material text). The complete dataset, *𝒟*, is composed of the set of all crossing events, *𝒟_X,I,E_*, by all individuals and in all experiments. Each model, *M_i_*, therefore specifies the probability of this dataset, conditioned on specified values for the free parameters *θ*, by multiplying over all these events. We follow the approach of [13,43,44] by integrating over the unknown parameters to obtain the probability of the data conditioned only on the model, *P*(*𝒟/M_i_*), and select the model for which the data is most probable (see the electronic supplementary material text for details).

## Notes

All work was approved by the University of Sydney's ethics reference no. L04/9-2008/1/4877.
